# Helminths of free-ranging dogs and cats in an urban natural reserve in Mexico City and their potential risk as zoonotic agents

**DOI:** 10.1371/journal.pone.0310302

**Published:** 2024-09-16

**Authors:** Valeria Camacho-Giles, Yolanda Hortelano-Moncada, Gerardo Torres-Carrera, Guillermo Gil-Alarcón, Alejandro Oceguera-Figueroa, Luis García-Prieto, David Osorio-Sarabia, Fernando A. Cervantes, Pablo Arenas

**Affiliations:** 1 Departamento de Zoología, Colección Nacional de Mamíferos, Instituto de Biología, Universidad Nacional Autónoma de México (UNAM), México City, México; 2 Departamento de Zoología, Colección Nacional de Helmintos, Instituto de Biología, Universidad Nacional Autónoma de México (UNAM), México City, México; 3 Secretaría Ejecutiva de la Reserva Ecológica del Pedregal de San Ángel, Universidad Nacional Autónoma de México (UNAM), México City, México; National Veterinary Research Institute (NVRI), NIGERIA

## Abstract

In the Reserva Ecológica del Pedregal of San Ángel, located in the south of Mexico City, Mexico, free-roaming dogs and cats coexist with 148 bird, 33 of mammal, 23 of reptile and seven amphibian species, that represent a remnant of the original fauna of the Mexican Plateau. The negative impact that dogs and cats have on local fauna is unobjectionable, however, the role that these introduced vertebrates play as potential transmitters of infectious diseases for native fauna and humans, is much less understood. Information about parasitic infections in native and introduced animals in this location is scarce. In order to ameliorate this lack of information, the objective of this study is to characterize the helminth fauna of the free-ranging dogs and cats of the ecological reserve. Between 2018 and 2023, 36 *Felis silvestris catus* and 7 *Canis lupus familiaris* were studied from the helminthological perspective. Endoparasites were obtained from the digestive tract and were identified to the species level using morphological and molecular evidence. Hosts were parasitized by eight species of helminths: in cats the cestodes *Hydatigera taeniaeformis*, *Mesocestoides* sp., *Taenia rileyi* and the nematode *Toxocara cati* were recorded, while in dogs, the cestode *Taenia pisiformis* and the nematodes *Ancylostoma caninum*, and *Uncinaria stenocephala* were found. The only species shared between cats and dogs was the cestode *Dipylidium caninum*. These free-ranging animals act as definitive hosts of 5 species known to have zoonotic potential; their presence in the area may generate a public and animal health problem if programs of dog and cat population control are not continued.

## Introduction

The Reserva Ecológica del Pedregal de San Ángel (REPSA) belongs to the Universidad Nacional Autónoma de México (UNAM). REPSA is a large-natural ecosystem completely surrounded by urban development within one of the largest and most densely inhabited cities of the world: Mexico City. REPSA is inhabited by native wildlife and free-ranging animals, that is, those that were abandoned or were born in the abandoned category or live free to roam [1]. Free-ranging animals do not receive food or medical attention [2] and represent a potential risk, as they develop wild behavior, becoming opportunistic animals and skilled predators that hunt wild animals in the areas they invade [3,4]. In addition to be threatened by free-roaming dogs and cats, native fauna is also exposed to the loss and fragmentation of its habitat [5,6]. In 2008, it was estimated that around 400 free-ranging cats lived within the REPSA. In addition, these authors pointed out that each year, between 40 and 80 newly abandoned, lost or stray dogs join to the pre-existing population. With the intention of reversing this situation, free-ranging fauna remediation programs were implemented between 2012 and 2016, in which 127 dogs and 41 cats were captured within the REPSA [6]. Free-ranging animals play an important role in the ecology, distribution and spread of diseases [3]. Their negative impact on the conservation of native biodiversity is amplified by the potential transmission of diseases, since they act as hosts for several groups of organisms, including virus, bacteria, protozoa and helminths, some of which may be zoonotic [2,7].

Due to the lack of information on helminths that affect dogs and cats in the wild in REPSA, the objective of the present study was to investigate their diversity, in order to characterize the composition and detect species with zoonotic potential.

## Materials and methods

The REPSA (19°18’21”; 19°20’11 N and 99°10’15”; 99°12’4” W), is an ecosystem known as “palo loco” xeric scrubland, within a highly urbanized area and represents one of the last relicts of the Pedregal Ecosystem south of Mexico City, valuable for conserving high biodiversity in the Mexican Plateau [6]. The total area of the REPSA is 237 ha, occupying approximately one third of the main UNAM campus. It is composed of 3 core zones (171 ha) and 13 buffer zones (66 ha) [8].

Dogs and cats analyzed in this study were obtained through the Authorization for management and control of exotic species, issued by Secretaría de Medio Ambiente y Recursos Naturales (SEMARNAT) to the personnel of the REPSA, under the permits of 09/F0-0079/06/18 and SGPA/DGVS/07077/21, 09/F0-0340/05/21. All animals studied were euthanized using the humanitarian methods established according to NOM-033-SAG/ZOO-2014 [9]. In brief, mammals (36 cats and 7 dogs) were anesthetized with a mix of ketamine (3–5 mg/kg) and xylazine (0.2–1 mg/kg), and euthanized with an overdose of intracardiac sodium pentobarbital (120–150 mg/kg); some specimens were kept frozen until dissection and others were studied freshly slaughtered. The gastrointestinal tract was examined comprehensively; the stomach and intestines were incised longitudinally and analyzed under the stereoscopic microscope. The collected helminths were washed in 0.85% saline solution and fixed in hot 70% alcohol. The specimens were separated in morphotypes and at least one of each morphotype was selected for molecular studies, by cutting a 5 mm tissue portion from proglottids region in the case of cestodes and midbody region in the nematodes. Finally, the specimens were stored in vials with alcohol at different percentages, according to the study to which they were subsequently subjected, i.e., 70% for morphology and absolute alcohol for molecular studies. Helminths were processed for their study following Lamothe-Argumedo [10]. Briefly, specimens used for morphological studies were stored in 70% alcohol. Cestodes were stained with Meyer’s paracarmin, Delafield’s hematoxylin, and Gomori’s trichrome, cleared with methyl salicylate, and mounted in permanent preparations with Canada balsam. Some scolices were artificially digested with pepsin-HCl mix to determine specific characteristics of the hooks. The nematodes were cleared with glycerin and Amann’s lactophenol. All the measurements are given in μm unless otherwise indicated, including the interval, with the average, standard deviation and sample size in parentheses.

The specimens studied under scanning electron microscopy (SEM) were stored in 100% alcohol and subsequently dehydrated to the critical point with CO2, using a K850 Critical Point Drier (Emitech, Ashford, England), then coated with a gold/palladium mixture with Q150R modular Coating System (Ashford, England), and examined at 15 kV in a Hitachi SU1015 SEM (Hitachi, Tokyo, Japan) at the Laboratorio Nacional de la Biodiversidad (LANABIO), Instituto de Biología, UNAM (IB-UNAM)

Representative specimens of each helminth species were subjected to molecular procedures, using a 2 mm of helminth tissue to extracting total DNA using the INVITROGEN Kit according to the manufacturer’s instructions; subsequently, the mitochondrial Cytochrome C Oxidase Subunit 1 (*cox1*), and the nuclear Internal Transcribed Spacer (ITS) and Large subunit of ribosomal DNA (28S rDNA) regions were amplified using the PCR with primers indicated in the Table 1; each reaction consisted of 9.5 μl of H2O, 3 μl of 5X buffer, 0.2 μl of each primer, 0.1 μl of Taq polymerase, and 2 μl of DNA, totaling a volume of 15 μL. When we used additional pairs of primers (as with NemF1–F3 and NemR1–R3) we diminished 0.2 μL of H2O for each extra primer. The general PCR thermocycling parameter included a denaturation at 94°C for 5 min, followed by 38 cycles of 94° C for 30s, 51°C for 30s, and 72°C for 1 min and a final extension period of 5 min at 72°C. The annealing stage was modified according to the loci to be amplified Table 1. Products of PCR were visualized in a 1.4% agarose gel. Successful amplifications were purified using CentriSep 96 filter plates (ThermoFisher Scientific, Pittsburgh, Pennsylvania) with Sephadex G-50 (Cytiva, Marlborough, Massachusetts). Sequencing reactions were composed of 0.4 μL BigDye.Terminator v.3.1 (Applied Biosystems, Waltham, Massachusetts, USA), 2 μL 5 × buffer, 4 μL ddH2O, 1 μL 10 μM primer and 3 μL purified PCR product (total volume 10 μL). Samples were purified using Sephadex G-50, then 25 μl de EDTA 0.5 mM was added to each sample and finally sequenced in an ABI-PRISM 3100 (Applied Biosystems® Waltham, Massachusetts) sequencer at LANABIO.

**Table 1:** Primers used for the amplification and sequencing procedures including the temperature parameters used in the annealing stage for the genes used for the molecular identification of helminth taxa obtained in free-ranging fauna from the REPSA, UNAM.

Locus	Primer	Sequence	Reference	Annealing temperature	Taxa
*cox1*	JB3	5’ TTTTTTGGGCATCCTGAGGTTTAT3’	[11]	51°C 40s	Cestoda
	JB4.5	5’ TAAAGAAAGAACATAATGAAAATG3’			
	PBI-COX1F_PCR (697–717)	5’ CATTTTGCTGCCGGTCARCAYATGTTYTGRTTTTTTGG3’	[12]		Cestoda
	PBI-COXIR_PCR (1280–1294)	5’ CCTTTGTCGATACTGCCAAARTAATGCATDGGRAA3’			
	NemF1_t1	5’ tgtaaaacgacggccagtCRACWGTWAATCAYAARAATATTGG3’	[13]	45°C40s (5x)– 51°C 40s (35x)	Nematoda
	NemF2_t1	5’ tgtaaaacgacggccagtARAGATCTAATCATAAAGATATYGG3’			
	NemF3_t1	5’ tgtaaaacgacggccagtARAGTTCTAATCATAARGATATTGG3’			
	NemR1_t1	5’ caggaaacagctatgactAAACTTCWGGRTGACCAAAAAATCA3’			
	NemR2_t1	5’ caggaaacagctatgactAWACYTCWGGRTGMCCAAAAAAYCA3’			
	NemR3_t1	5’ caggaaacagctatgactAAACCTCWGGATGACCAAAAAATCA3’			
28S	28z (28dd) (1129–1154)	5’ AGACTCCTTGGTCCGTGTTTCAAGAC3’	[14]	53°C 35s	Cestoda Nematoda
	28y (28cc) (67–96)	5’ CTAACCAGGATTCCCTCAGTAACGGCGAGT3’			
	501	5’ –TCGGAAGGAACCAGCTACTA3’	[15]	55°C45s	Cestoda Nematoda
	504	5’ –CAAGTACCGTGAGGGAAAGTTG3’			
ITS	BD1	5’ –GTCGTAACAAGGTTTCCGTA3’	[11]	55°C45s	Cestoda Nematoda
	BD2	5’ TATGCTTAAATTCAGCGGGT3’			

Identification of cestodes on the morphological basis was conducted following Khalil et al. [16], and for nematodes, Anderson et al. [17]. In addition, the original description of each species was consulted. Voucher specimens of each helminth were deposited in the Colección Nacional de Helmintos (CNHE), IB-UNAM, and the DNA sequences are available in GenBank. The characterization of the infections with the parameters: Prevalence, Mean Abundance, Mean Intensity and Intensity range, was carried out according to Bush et al. [18]. Raw data associate to this work are available in a (S1 Table).

## Results

Of the 43 free-ranging animals examined (36 cats and 7 dogs), all collected between 2018 and 2023, 21 cats and 7 dogs were found parasitized with helminths; the collected helminth species belong to the phylum Platyhelminthes and Nematoda. In cats, the cestodes *Hydatigera taeniaeformis*, *Mesocestoides* sp., *Taenia rileyi* and the nematode *Toxocara cati* were recorded, while in dogs, the cestode *Taenia pisiformis* and the nematodes *Ancylostoma caninum*, and *Uncinaria stenocephala* were found. The cestode *Dypilidium caninum* is the only shared species among the cats and dogs examined, and reaches the highest prevalence in dogs, while the nematode *T*. *cati* was the most prevalent in cats. With the exception of *Mesocestoides* sp. in cats, both helminth mean abundance and mean intensity were higher in dogs Table 2.

**Table 2:** Infection parameters of helminths in free-ranging dogs and cats from the Reserva Ecológica del Pedregal de San Ángel in Mexico City, Mexico.

Helminths species	*Canis lupus familiaris*				*Felis silvestris catus*			
Cestoda	Prevalence	Mean abundance	Mean intensity	Intensity range	Prevalence	Mean abundance	Mean intensity	Intensity range
*Dipylidium caninum*	71.42	8.28	11.6	3–25	19.44	1.58	8.14	1–21
*Hydatigera taeniaeformis*					19.44	0.47	2.42	1–6
*Taenia pisiformis*	57.14	20.57	36	1–138				
*Mesocestoides* sp.					19.44	17.86	91.85	1–450
*Taenia rileyi*					11.11	0.19	1.75	1–4
**Nematoda**								
*Toxocara cati*					50	4.58	9.16	1–26
*Ancylostoma caninum*	42.85	4.85	11.33	5–22				
*Uncinaria stenocephala*	28.57	5.42	19	17–20				

Based on the morphological and molecular studies carried out, the diagnostic characteristics of each of the 8 species found are presented below; briefly discuss the criteria followed for their identification:

Cestoda

Taeniidae

### *Hydatigera taeniaeformis* Batsch, 1786

**Material:** CNHE: 11833, 13003. *Cox1* GenBank: OQ281682, OQ281678, PP574870; 28S GenBank: OQ413990.

**Fig 1:**
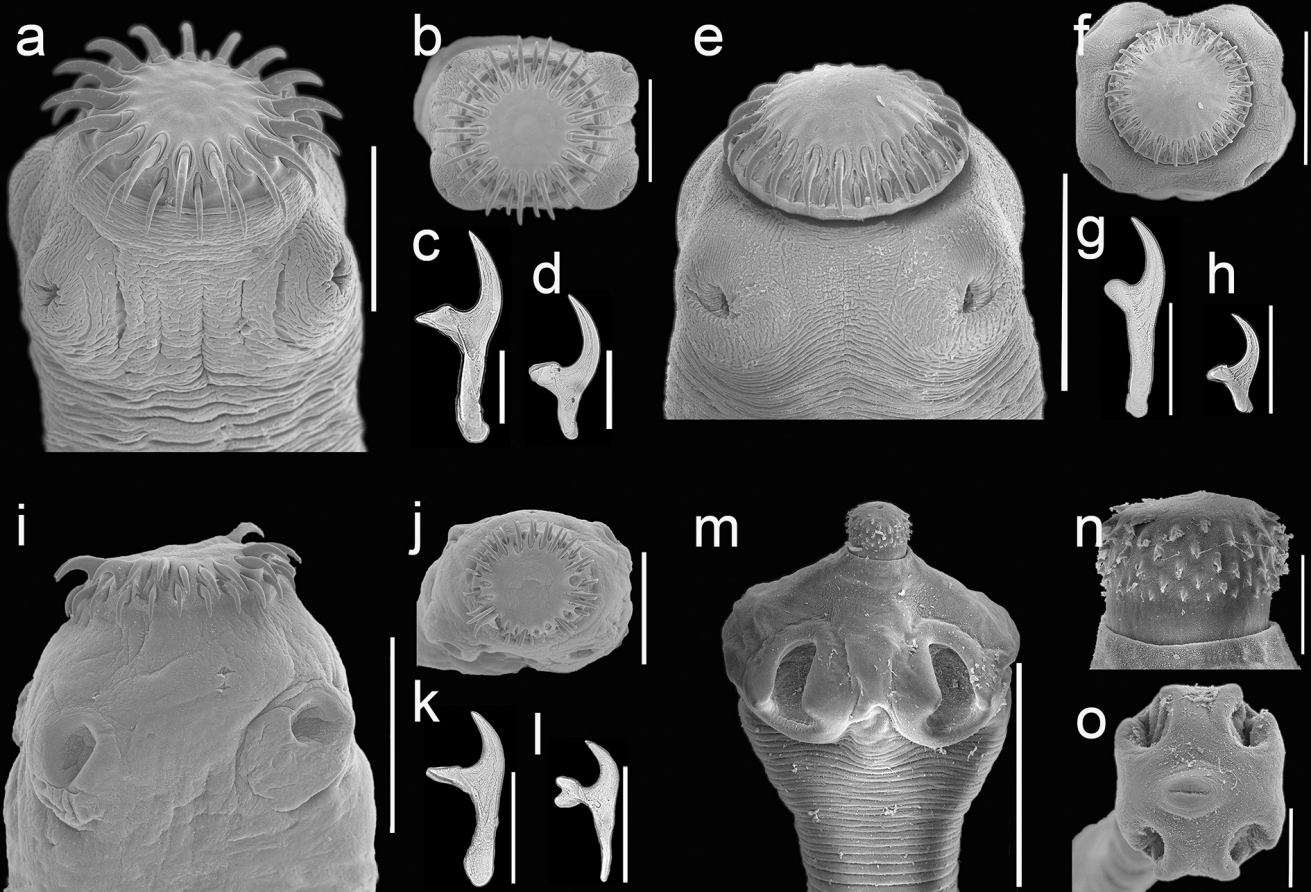
Scanning electron microscopy and light micrographs of cestodes found in the gastrointestinal tracts of free-ranging dogs and cats from the REPSA. a) *Hydatigera taeniaeformis* scolex; b) apical view; c and d, rostellar hooks; e) *Taenia pisiformis*, scolex; f) apical view; g and h, rostellar hooks; i) *Taenia rileyi* scolex; j) apical view; k and l, rostellar hooks; m) *Dipylidoium caninum* scolex; n) armed rostellum; o) scolex with invaginated rostellum.

**Diagnosis:** Based on 15 individuals. Scolex with armed rostellum with 2 crowns of 30–40 hooks (35.5 +/- 4.43; n = 5), larger hooks 306–430 (420 +/- 0.017; n = 7; 52 hooks); smaller hooks 250–270 (270 +/- 0.010; n = 6; 57 hooks) in length (Fig 1A–1D). Number of testes in mature proglottids 278–544 (384.6+/- 81.94; n = 6; 15 proglottids).

**Remarks:** Specimens were identified based on the number and size of large and small hooks in the 2 crowns of the rostellum, according to Esch and Self [19] for canids and felids from USA; Panti-May et al. [20,21] for rodents from Mexico, and Lavikainen et al. [22] for rodents and cats from several countries around the world. Blastn search of *cox1* gene results in identical sequences to *H*. *taeniaeformis* from Peru (OQ569222-OQ569229) found in rodents [23]. Likewise, there is a relatively high similarity (up to 98%) with the rest of the sequences published in GenBank from other parts of the world [22] corroborating the global occurrence of this species.

### *Taenia pisiformis* Bloch, 1780

**Material:** CNHE: 13001–2. *CoxI* GenBank: PP472462; 28S GenBank: PP574874.

**Diagnosis:** Based on 11 specimens with scolex with two crowns of 42–46 hooks (43.82 +/- 1.40; n = 11) on the rostellum; hooks of anterior row 240–260 (250 +/- 0.0060; n = 11; 129 hooks) in length, and hooks of the second row 140–150 (150+/- 0.0057; n = 11; 125 hooks) in length (Fig 1E–1H). Number of testes in mature proglottids 200–366 (275.77+/- 50.08; n = 11; 13 proglottids). Gravid proglottids with 14–24 (19.17 +/- 3.43; n = 3; 6 proglottids) uterine branches.

**Remarks:** The morphologic traits of rostellar hooks, which number and size are in accordance with those recorded by Riser [24] for this cestode parasitizing felids from USA; Verster [25] and Loos-Frank [26] for different species worldwide and by Esch and Self [19] for canids and cats from USA, allowed the identification of this species. Blastn search resulted in low similarity with the sequences previously published for both *T*. *pisiformis* and other species of *Taenia*, with similarities ranging from 88% to 91% with 28S locus. In the same way, *cox1* blastn search resulted in the highest similarity (92.04%) with a sequence obtained from a cysticercus of *T*. *pisiformis* from Poland (MZ287426) found in a rabbit [27]. The wide genetic variation detected in this study suggests that this cestode species may represent a complex of more than one species, or alternatively, that the DNA sequence of *T*. *pisiformis* available in Genbank are the result of a morphological misidentification.

### *Taenia rileyi* Loewen, 1929

**Material:** CNHE: 12997–98. *Cox1* GenBank: PP574871.

**Diagnosis:** Based on 7 scolices with two rows of 34–46 (41.33 +/- 7.0710; n = 5) hooks on the rostellum, larger hooks 220–240 (230 +/- 0.00538; n = 6; 46 hooks), and smaller hooks 150–180 (170 +/- 0.00885; n = 4; 43 hooks) (Fig 1I–1L).

**Remarks:** The number of hooks and their measurements fit with the wide variability range reported for this species by different authors in several hosts throughout their distribution; according to the above, the number of hooks reported for *T*. *rileyi* ranges from 36 to 46; the size of the largest ones oscillates from 205–258 and the smallest ones from 151 to 205 [21,24,26,28,29]. A blastn search recovered *Taenia* sp. as the first match with 90.38% of similarity, with specimens recovered from wild cats from Colombia (MZ351293) [30]. Sequences generated in the present study represent the first *cox1* sequence for this species of cestode.

Dipylidiidae

### *Dipylidium caninum* (Linnaeus, 1758) Leuckart, 1863

**Material:** CNHE 10977, 11875. *Cox1* GenBank: OQ281679; 28S GenBank: OQ401031

**Diagnosis:** Based on 21 specimens with scolex with a protrusible rostellum armed with 75–90 (84 +/-7.93; n = 7) hooks, distributed in 3–6 rows, hooks length between 0.006–0.015 (11 +/- 0.003; n = 3; 146 hooks) (Fig 1M–1O). Number of testes in mature proglottids 103–277 (195.62 +/- 42.66; n = 11; 71 proglottids); two sets of genitalia. Gravid proglottids contain numerous ovigerous capsules with 4–15 eggs each (7.69 +/- 2.08; n = 9; 31 capsules); eggs diameter ranging from 1–3 (1.90 +/- 0.91; n = 9; 155 eggs).

**Remarks:** Adults specimens were identified on the basis of the features of rostellum, such as having uniformly thorn-like hooks distributed in several rows, vaginal opening posterior to cirrus sac and because each ovigerous capsules contain several numbers of eggs [16]. These characteristics have also been referred as diagnostic for the species by Venard [31] in parasites from dogs and cats from USA; for dogs, cats and humans from Mexico [32,33]; India [34–36]; China [37] and Portugal [38]; Blastn search of *cox1* gene resulted in identical sequences for *D*. *caninum* from Italy (MT806359) found in red foxes [39]. In the same way, 28S gen query resulted in identical sequences to *D*. *caninum* from several localities of Europe, South Africa and USA (MH040824- MH040861, MH045472- MH045481) found in dogs and cats [40].

Mesocestoididae

### *Mesocestoides* sp.

**Fig 2:**
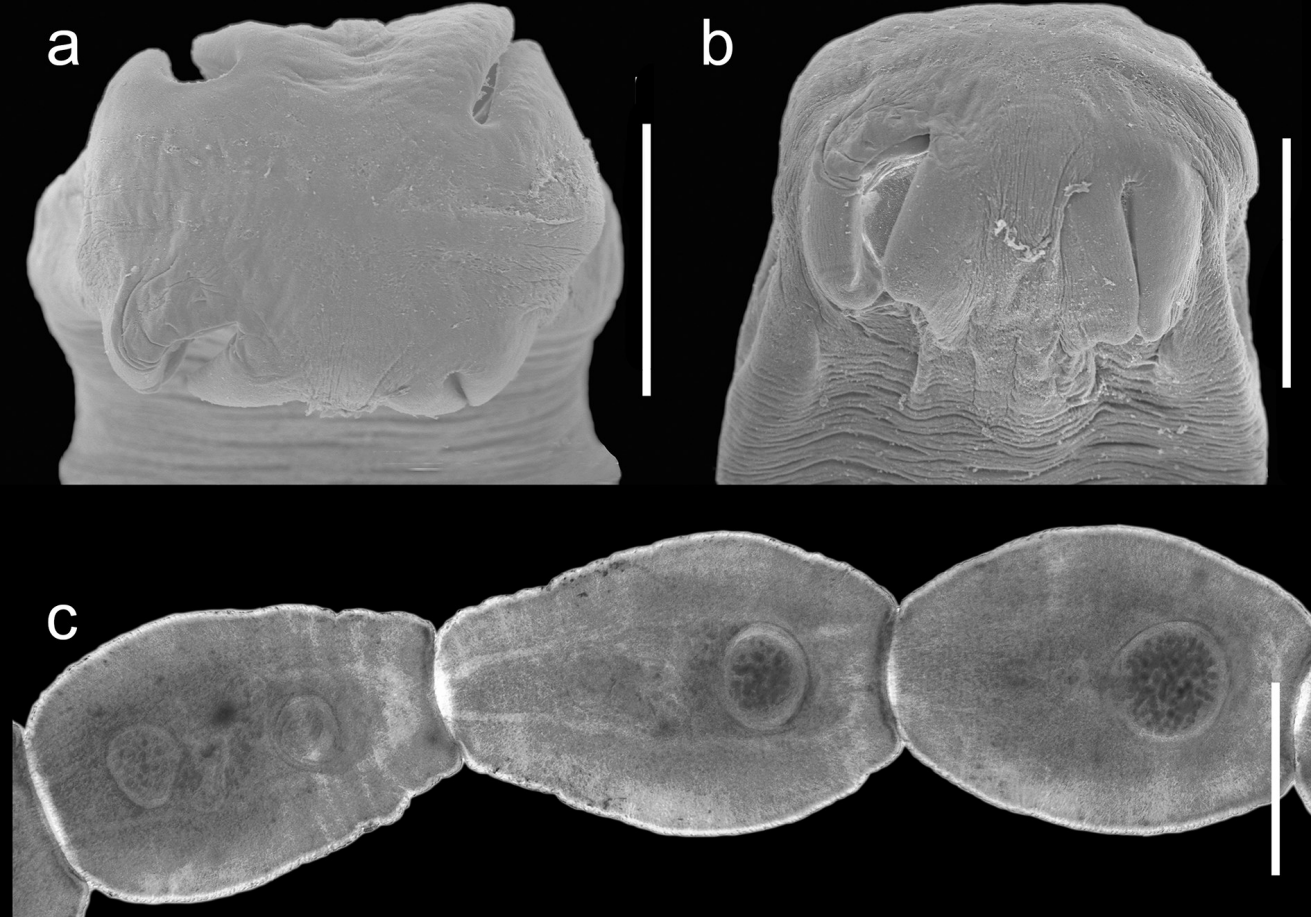
Scanning electron microscopy and light micrographs of *Mesocestoides* sp. found in the gastrointestinal tract of free-ranging cats from the REPSA. a) Scolex, apical view; b) ventral view; c) gravid proglottids (stained slides).

**Material:** CNHE: 12995–6. *Cox1* GenBank: OQ281680, OQ281681; 28S GenBank: OQ343502. (Fig 2).

**Diagnosis**: Based on 15 unarmed scolices and 26 proglottids. Scolex with 4 round suckers (Fig 2A and 2B). Mature proglottids contain 38–51 (46.12 +/- 4.55; n = 8) testes. Gravid proglottids with paruterine organ [(0.273–0.464 (0.35+/-0.05; n = 15) length by 0.209–0.382 (0.30+/-0.04; n = 15) width, and 0.018–0.046 (0.03+/-0.01; n = 15) wall thickness]. Central genital pore.

**Remarks:** Adult worms collected in free-ranging cats present morphological features diagnostic of *Mesocestoides* such as cirrus-sac and vaginal duct opening in a genital atrium toward ventral surface of proglottid midline; vitelline gland bilobed, and uterus replaced by a single paruterine organ, according to Khalil et al. [16] and Caira and Jensen [41]. According to Caira et al. [42], four species of *Mesocestoides* are valid: *M*. *ambiguous* Vaillant, 1863 from Africa ex. *Vivera genetta*; *M*. *corti* Hoeppli, 1925 ex. *Mus musculus* from the USA; *M*. *melesi* Yanchep and Petrov, 1985 ex. *Meles* sp. from Bulgaria and *M*. *vogae* Edges 1991 ex. *Sceloporus occidentalis* from USA. In Mexico, only 2 species have been described: *M*. *vogae* ex *Canis lupus familiarias* [43] and *M*. *bassarisci* McCallum 1921 ex *Basssariscus astutus* [44], but the validity of the latter has not been confirmed [42].

The *cox1* and 28S sequences blast analyses resulted in 91.47% and 97.07% of identity, respectively, with sequences deposited in GenBank for species of this genus that parasitize rodents (NC_061204) from China [45], and domestic mammals (MK239661) from Europe and Africa [46], respectively. Except for *M*. *ambiguous*, the sequences of three of the species mentioned above have been deposited in GenBank, but none significantly match the sequences generated here.

Nematoda

Ascarididae

### *Toxocara cati* (Schrank, 1788) Brumpt, 1927

**Material:** CNHE: 11832. ITS GenBank: OQ256234; 28S GenBank: OQ343501

**Fig 3:**
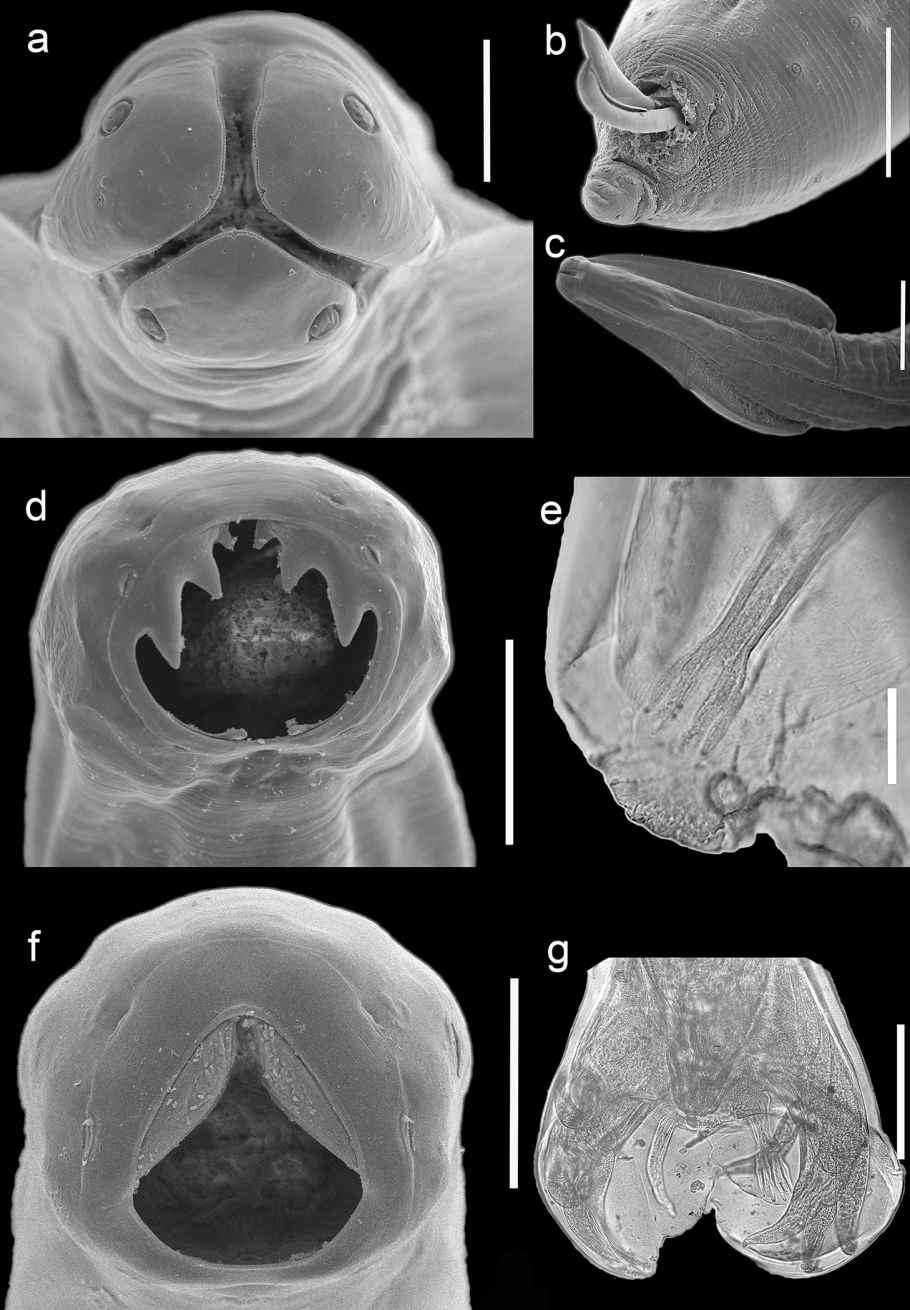
Scanning electron microscopy and light micrographs of nematodes found in the gastrointestinal tract of free-ranging dogs and cats from the REPSA. a) *Toxocara cati*, lips, apical view; b) posterior end of male, showing spicules; anterior end, ventral view, showing lateral alae. d) *Ancylostoma caninum*, buccal capsule, showing teeth; Copulatory bursa, showing dorsal ray with 2 large and one short terminal prongs (cleared with lactophenol); f) *Uncinaria stenocephala* buccal capsule showing chitinous plates; Copulatory bursa, showing dorsal ray with 3 terminal prongs of the same size (cleared with lactophenol).

**Diagnosis**: Based on 28 specimens (15 females and 13 males). Both sexes with a pair of cervical alae, which give the anterior end of the body an arrow-like appearance. Mouth located anteriorly with three lips, one large dorsal and two smaller ventro-lateral. Dorsal lip has two large papillae; each ventrolateral lip has only one. Dentigerous ridges arranged on the margin of each lip. Females: body 4.5–8.2 mm (6.9 mm +/- 1.21; n = 12) long by 0.88–2.04 mm (1.33 mm +/-0.36; n = 12) wide; distance between the anus and the anterior end of the body 0.43–0.64 mm (0.56 mm +/-0.07; n = 12). Males: body 3.5–6 mm (5.2 mm +/- 0.79; n = 11) long by 0.81–1.48 mm (1.21 mm +/-0.22; n = 11) wide. Genital papillae, distributed as follows: 12–22 (17.75 +/-3.28; n = 12) precloacals, 2 (n = 12) adcloacals and 4 (n = 12) postcloacals. Paired spicules, 1.97–2.68 mm (2.19 mm +/- 0.22; n = 13) (Fig 3A–3C).

**Remarks:** Adult worms collected in the free-ranging cats studied were identified at specific level based on the features in anterior and posterior ends of body in males and females. Anterior section lips well-defined in both sexes, a pair of spicules and several cloacal papillae in posterior end of males. All these traits agree with those presented in the re-description of the species made by Sprent [47] for parasites of cats from Australia; Gallas and Fraga [48] from wild felines from Brazil; Radwan et al. [49] for canids and felines from Egypt and Mekete [50] for cats from South Africa. Blastn query of 28S sequence results in a similarity of 99.51% with specimen found in wild cats from China (JN256994) [51], and 100% for ITS (MF592398) with a specimen from Iran [52] found in soil samples with eggs of *Toxocara*.

Ancylostomatidae

### *Ancylostoma caninum* (Ercolani, 1859)

**Material:** CNHE: 12999. *Cox I* GenBank: OQ290602; ITS GenBank: OQ256235, OQ256236.

**Diagnosis**: Based on 34 individuals (21 females and 13 males). Mouth opening with a pair of prominent chitinous plates provided with three pairs of ventral teeth. Females: body 0.4–1.5 mm (1.11 mm +/- 0.28; n = 20) length by 0.26–0.63 mm (0.49 +/- 0.11; n = 19) wide; vulva at the posterior end of the body, which ends in a spine; distance between anus and tail 0.15–0.25 mm (0.21 mm +/-0.03; n = 19). Males: body 0.2–1.1mm (0.96 mm +/- 0.25; n = 11) length by 0.29–0.50 mm (0.39 mm +/-0.05; n = 12) wide. Body ending in a copulatory bursa; ventrolateral and lateroventral rays fused for one-half length from origin in base of bursa; lateral rays with a common stem; externo-dorsal rays bifurcate from base of dorsal ray, forming two lateral lobes. Dorsal ray divided into two branches with each branch terminating in three digitations different in size; spicule equal in size, 0.73–0.96 mm (0.77 mm +/- 0.10; n = 10) (Fig 3D and 3E).

**Remarks:** Adult worms collected from the free-ranging dogs studied here were identified at specific level based on the characteristics of the anterior and posterior ends of the body of both sexes, mainly in the buccal capsule armed with three pairs of ventrolateral teeth, and the dorsal rays of the bifurcated copulatory bursa, features that agree with those presented in the re-description of the species by Burrows [53] for cats and dogs from USA, and Uppal et al. [54] for dogs from India. Blastn query of ITS gen results in a similarity of 100% with specimens found in dogs and cats from Australia (KP844730) [55] and 100% with *cox1* gen with a specimen found in gray fox *(Urocyon cinereoargenteus)* from Mexico (MZ821647) [56].

### *Uncinaria stenocephala* (Raillet, 1884) Froelich, 1789

**Material:** CNHE: 13000. *Cox 1* GenBank: PP574872, PP574873; 28S GenBank: PP472461

**Diagnosis:** Based on 38 specimens (20 females and 18 males). Buccal capsule distinguished by the presence of a pair of chitinous lateral plates with rounded margins. Females: body 0.7–1.1 mm (0.84 mm +/- 0.110; n = 19) long by 0.26–0.36 mm (0.31mm +/-0.030; n = 7) wide; distance between vulva and anterior end of body 5.01–7.23 mm (5.95 mm +/- 0.98; n = 4), and between anus and tail 0.16–0.23 mm (0.19 mm +/- 0.018; n = 19). Males: body 0.6–0.9 mm (0.78 +/- 0.098; n = 18) long by 0.30–0.25 mm (0.28 +/-0.019; n = 9) wide. Copulatory bursa with semi-ovate lateral lobes; dorsal ray distally forked, each branch ending in three pronged of similar size. Spicules of equal size 0.76–0.68 mm (0.70 mm +/-0.047; n = 13) (Fig 3F and 3G)

**Remarks:** Adult worms collected from the free-ranging dogs studied were identified at a specific level based on the characteristics at the anterior and posterior ends of the body in males and females, traits that agree with those presented in the re-description of the species carried out by Górski et al. [57] for canids from Poland and Ransom [58] for dogs, foxes and badgers from USA. The sequence obtained for the 28s gene had 100% similarity compared to larvae of this nematode species (MT343056) by Karadjian [59] from France. The sequences of *cox1* gene generated in our study represent the first for this nematode species.

## Discussion

Invasion ecology occupies an important role in conservation biology, because invasive species have become the second most important cause of species loss [60]. The host species studied here are considered invasive and have been spread out worldwide and are living in constant interaction with native animals (mammals, birds, reptiles, etc.), which favors the transmission and exchange of parasites and other pathogens [60].

In Mexico [56] and elsewhere [39,45,51], the presence of parasites, such as *D*. *caninum*, *A*. *caninum*, *T*. *cati* and *Mesocestoides* sp., in wildlife has been reported, which suggests that the native vertebrates of the REPSA may be parasitized by some of the species that we have recorded in dogs and cats although to date there are no systematic studies that confirm this.

A considerable number of species of intestinal parasites, whose definitive hosts are dogs and cats, have the capacity to infect humans [61]. According to Salyer et al. [62] and Rhaman et al. [63], about 60% of emerging human infectious diseases have a zoonotic origin. Among neglected tropical diseases, Xiao et al. [64] and Sapp and +Bradbury [65] consider zoonotic helminthiasis as the most common human pathogens; these authors estimate that they represent a greater global burden of disease than infections such as malaria and tuberculosis. Based on the results obtained in this study, five of the eight helminth species identified (*D*. *caninum*, *Mesocestoides* sp., and the three nematode species) have zoonotic potential [66]. Transmission routes followed by helminths to infect their definitive hosts are variable, since it can be through the direct ingestion of eggs or larvae allocated in intermediate hosts (in the case of cestodes and other helminths) or, in nematodes by skin penetration, vectors and in general, by environmental contamination [32]. As is known, the 5 species of cestodes that we report here were recruited by cats and dogs through the ingestion of intermediate hosts such as arthropods (in dipylidiasis), rodents and lagomorphs (for the taeniids) and vertebrates in general (in the case of *Mesocestoides* sp.) [41].Two of the three nematode species (*A*. *caninum* and *U*. *stenocephala)* infect dogs percutaneously while *T*. *cati* enter cats by ingestion of eggs or transmammary [17]. Humans can be accidentally infected with these helmints through the same routes, except for the transmammary route. However, despite not being the natural host of these helminths, diseases caused by these species can have an impact on public health. For example, Rostami et al. [67] estimated the global seroprevalence for human toxocariasis to be 19%. For infections caused by hookworms, values of 32% have been recorded worldwide. Garcia-Agudo et al. [68] conducted a review of cases of dipylidiasis in children worldwide, reporting 16 cases in the last 20 years since 1994. In the case of *Mesocestoides* spp., according to Fuentes et al. [69] 27 cases in human have been reported in six countries.

Taeniidae is made up of a wide variety of species, many of which can cause infections in humans; however, species reported in this study (*T*. *pisiformis*, *H*. *taeniaeformis* and *T*. *rileyi*) have not been registered as zoonotic, because the intermediate hosts involved in their biological cycles are not included in the human diet in regular conditions [70].

Most of the studies in human populations detect helminth infections through coproparasitoscopic examinations, so their level of precision in terms of the taxonomic identity of the parasites is not high, particularly in hookworms and taeniids. This highlights the importance of confirming the species identity of helminth through various sources of information; particularly the availability of DNA sequences associated with robust morphological identifications, as they facilitate diagnosis by reducing the margins of error.

In conclusion, the potential zoonotic disease risks posed by dogs and cats within REPSA provide additional support for continuing control programs; likewise, it is necessary to raise awareness in society about the damage that harmful fauna (dogs and cats) causes to native species and the risk of transmission of parasitic infections to humans. In this sense, both dogs and cats can be transmitters of other pathogens such as bacteria [71], viruses [3] and arthropods [72]. The lack of awareness of these major issues in the general public and by decision makers prevents the control of parasitic zoonosis, since allegations of mistreatment and cruelty towards domestic animals are common [73]. In reality, free-ranging animals cause extensive damage to the ecosystem including to native animals to these ecosystems, so a more holistic view of the effect of the removal of free-ranging animals needs to be consider by these parties. Ramos-Rendón et al. [5] demonstrated how effective control programs of these invasive animals are in the REPSA; after their application of these programs, they determined not only the increase in some populations of native animals, but also the reappearance of others such as the gray fox (*U*. *cinereoargenteus*), who had no records in the area for years. The disclosure of the findings of this works is relevant to warn the general public about the consequences of abandoning domestic fauna in natural ecosystems, particularly in urban reserves like the REPSA, which is a highly anthropized environment where around 270,000 persons cohabit with these species every day [6].

## Supporting information

S1 Table Raw data of helmints recovered of free-ranging dogs and cats from the Reserva Ecológica del Pedregal de San Angel. (XLSX)
